# Biodiversity data rescue in the framework of a long-term Kenya-Belgium cooperation in marine sciences

**DOI:** 10.1038/s41597-019-0092-8

**Published:** 2019-06-12

**Authors:** Carolien Knockaert, Lennert Tyberghein, Annelies Goffin, Delphine Vanhaecke, Harrison Ong’anda, Enock O. Wakwabi, Jan Mees

**Affiliations:** 1 Flanders Marine Institute, Wandelaarkaai 7, 8400 Oostende, Belgium; 2 Kenya Marine and Fisheries Research Institute Mombasa Centre, P.O. Box 81651, 80100 Mombasa, Kenya

**Keywords:** Marine biology, Biodiversity

## Abstract

The Kenya-Belgium data collection includes about 111,800 biotic observations on benthos, algae, fish, zooplankton, phytoplankton, birds and mangroves which cover more than 400 unique locations that were sampled between 1873 and 1999. The scope of this data digitization project was to recover data in theses and reports resulting from marine and coastal research activities in the Eastern African region conducted between 1984 and 1999. Data were digitized and quality checked in the frame of the Belgian LifeWatch project. The dataset provides a better insight into the different types of research conducted between 1985 and 1996 in frame of the Kenya-Belgium cooperation in marine sciences (KBP) project and can facilitate further coastal biodiversity research in Kenya.

## Abstract (summary)

**Table:** 

Design Type(s)	data integration objective • species comparison design • data normalization objective
Measurement Type(s)	Scientific Publication
Technology Type(s)	digital curation
Factor Type(s)	temporal_interval • Species • geographic location
Sample Characteristic(s)	Actinopterygii • Kenya • ocean biome • Acantharia • Asteroidea • Anthozoa • Chromadorea • Florideophyceae • Arachnida • Hexanauplia • Malacostraca • Ulvophyceae • Bacillariophyceae • Holothuroidea • Enoplea • Aves • Dinophyceae • Polychaeta • Leptocardii • Ophiuroidea • Cyanophyceae • Bivalvia • Appendicularia • Gastropoda • Ascidiacea • Echinoidea • Magnoliopsida • Rhabditophora • Cephalopoda • Branchiopoda • Insecta • Collembola • Crinoidea • Phaeophyceae • Thaliacea • Clitellata • Hydrozoa • Kinorhyncha • Larvacea • Loricifera • Scyphozoa • Ostracoda • Pycnogonida • Chlorophyceae • Priapulida • Sagittoidea • Dictyochophyceae • Phyllopharyngea • Spirotrichea • Turbellaria

Machine-accessible metadata file describing the reported data (ISA-Tab format)

## Background & Summary

The Kenya-Belgium cooperation in marine sciences (KBP) project was launched in 1985^1^ as an initial collaboration between the Free University of Brussels (VUB) and the Kenya Marine and Fisheries Research Institute (KMFRI) under the supervision of the late Prof. Dr. Philip Polk. The aim was to improve the collaboration between different scientific institutes and marine scientists in Belgium and Kenya. The project consisted of three successive phases. The first phase of the KBP, named Cooperation in the field of marine ecology and management of the coastal zone (1985–1988) included an update of the necessary sampling equipment (basic lab, cars, boats and computers) and the start of a postgraduate course Fundamental and Applied Marine Ecology (FAME) at VUB for scientists of developing countries. The first fundamental research efforts were logically directed at the inventory and description of fauna and flora at the Kenyan Coast (Gazi Bay). The initial phase was extended for another four years (1989–1992) in a new project called Higher institute for marine sciences. The main objective was to conduct fundamental research in the areas of plankton, reef ecology, water chemistry, coastal oceanography and modelling, fisheries, algae, pollution and library sources. The successful cooperation between Kenya and Belgium and the coordinating role of the KBP proved to be a major attraction to other countries in East-Africa and Europe and resulted in several major international projects. As a logical extension, a third phase of the KBP started with a project on Research towards sustainable exploitation of natural resources in mangrove forests (1992–1996) with the continuation of the research on fish, crustaceans, birds, algal and phytoplankton cultivation and pollution monitoring. Most important realizations in this phase were the development of African’s largest oyster farm in Gazi Bay and the reforestation of more than 10 hectares of mangrove forests (90,000 trees of 5 different mangrove species). Between 1985 and 1996, a total of 13 KMFRI researchers completed the FAME course at the VUB. During this period, the number of scientific publications, especially in international journals, increased considerably (Table 1). In 2012, the long lasting collaboration as described above resulted in a formal Memorandum of Understanding (MoU) between the Flanders Marine Institute (VLIZ) and the Kenya Marine and Fisheries Research Institute (KMFRI). This MoU aims to promote further partnership in the field of marine sciences between Belgium and Kenya (in a coordinated way). One of the activities within this MoU is the recovery of data resulting from marine research in Kenya. In the past, the data collected during the KBP were mostly scattered and even hidden in the publications and reports coming out of the project. In that way, such data are nowadays hard to find for researchers: a lot of time may be lost when figuring out either where to find the data in literature or what type of data exist in a particular scientific work. Thanks to this project potentially high interesting data were digitized, standardized, quality controlled and brought together in one comprehensive dataset. Overall, this data rescue project focused on the digitization and online disclosure of distribution records of observed taxa in both time and space to make these data accessible again for the scientific community. The Kenya-Belgium data collection is online available through the LifeWatch portal (http://www.lifewatch.be/en/marine-data-archeology) and can be assessed through the Integrated Marine Information System (IMIS) hosted at VLIZ.

**Table 1:** Scientific publications by KMFRI researchers between 1975 and 1994. Adapted from Seys, J.^1^.

Source	1975–1979	1980–1984	1985–1989	1990–1994
Theses	0	9	8	9
Local journals and local symposia	3	24	30	22
International journals	1	4	27	34
Proceedings intern. symp.	0	5	3	3
Total	4	42	68	68

## Methods

### Data inventory

The first step was to identify potential literature for digitization. The Belgian Marine Bibliography or BMB (http://www.vliz.be/en/belgian-marine-bibliography) was searched to set up a Kenyan literature collection. The BMB is a reference list of publications on the Flemish coast and the Belgian Part of the North Sea as well as other marine, estuarine and coastal publications written by Belgian authors and scientists or foreign scientists affiliated with Belgian institutes. The BMB query resulted in a total of 402 theses and reports. First authors and affiliated institutes were contacted to gain approval and to comment on the digitization of the data in each publication. After setting priorities (selection of all publications concerning marine research in Kenya and the Eastern African region and approval by original authors and/or Belgian institutes for digitization and disclosure of the data), a total of 70 publications (29 reports and 41 theses) were selected for data digitizing (Online-only Table 1).

### Quality control, standardization & data output

Data were digitized using a data format containing standard fields for a correct understanding of the data. The most important fields are the location linked through a Well-Known Text (WKT), sampling protocol and sample size, event date, scientific name, sex, lifestage and related measured biotic (describing the living organisms in the ecosystem) or abiotic parameters (chemical or physical parameters describing the environment of the observed species). Metadata were added by searching the original publications. The species taxonomy was verified using the taxon match tool offered by the World Register of Marine Species (WoRMS - http://www.marinespecies.org), an authoritative and comprehensive list of names of marine organisms edited and reviewed by an international team of more than 240 taxonomic editors world-wide. Every species has a unique identifier^2^ known as the AphiaID. This identifier links the species name to an internationally accepted standardized name and associated taxonomic information, and also redirects to the most accurate information on the species taxonomy, (e.g. accepted names and synonyms). Next to the taxonomic information basic trait information was added in the database using Marine Species Traits (http://www.marinespecies.org/traits). If not present, geographical coordinates were added to the sampling locations either by georeferencing in QGIS based on the maps present in the particular studies or by matching with the Marine Regions Gazetteer^3^. In the next step datasets were created and described in the Integrated Marine Information System (IMIS) and archived in the Marine Data Archive (MDA - http://mda.vliz.be). In the last step a Digital Object Identifier (DOI) was assessed to each dataset making them more visible, traceable and citable. A total of 86 datasets were finished and assigned with a DOI. Biotic datasets were transferred and integrated in AfroBIS (http://afrobis.csir.co.za), the African node of the Ocean Biogeographic Information System (OBIS) and can be downloaded though the EurOBIS (http://www.eurobis.org)/EMODnet Biology (http://www.emodnet-biology.eu) platform and via the KMFRI IPT (http://ipt.vliz.be/kmfri) hosted at VLIZ.

### Data selection

The Kenya-Belgium data collection focused on the temporal and geographical distribution of biotic data. Experimental data (e.g. cage exclusion experiments) and data on stomach analysis of fish species were excluded from the collection. Eventually 68 unique biota datasets were selected to integrate in the data collection (Online-only Table 2).

## Data Records

The Kenya-Belgium data collection contains a total of 111,784 biotic observations in the Eastern African region dating from 1873 till 1999.

### Geographical coverage

**Fig. 1:**
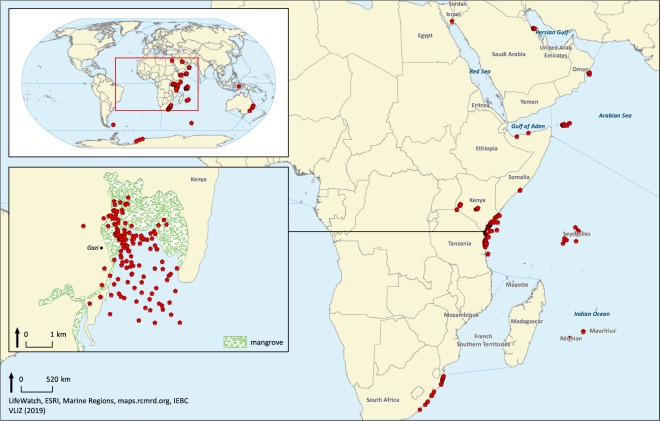
Georeferenced map of all biological observations and sampling locations at Gazi Bay, Kenya.

Samples were collected at 408 point locations (Fig. 1). Most of these were located along the Kenyan Coast, more specifically at Gazi Bay, and represented about 87% of database records. This is easily explained because the first research efforts made in frame of the KBP were to make an inventory and description of fauna and flora at the Kenyan Coast and Gazi Bay. The second largest collection of observations originated from the coastal area of Tanzania, followed by the East Coast of South Africa. Furthermore, a substantial part of the dataset observations came from the Seychelles and Socotra.

### Themes & temporal coverage

**Fig. 2:**
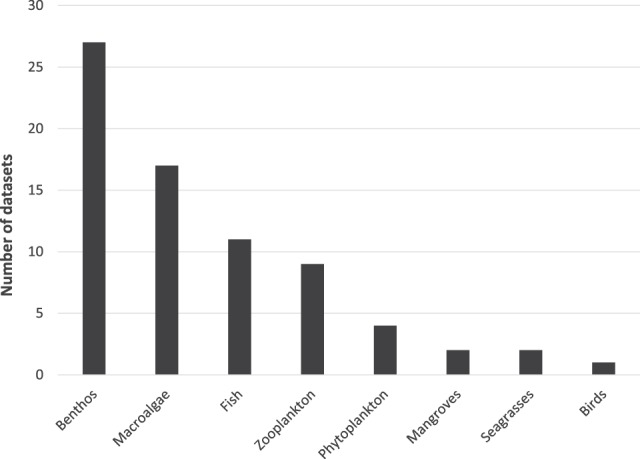
Thematic coverage of datasets.

**Fig. 3:**
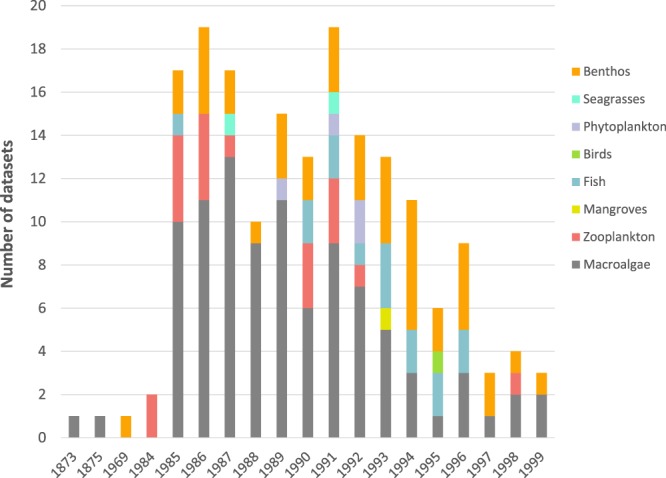
Temporal distribution of biological datasets per theme.

The biological datasets were classified into 8 groups based on the number of observations belonging to a specific functional group (lower limit 70%) (Fig. 2). Data are available from 1873 to 1999 (Fig. 3). Data records from 1873, 1875 and 1964 are literature records and were not part of research field surveys. The increase in number of datasets per year is clearly correlated with the launch of the KBP and the start of the FAME course for KMFRI researchers. The number of field surveys increased immediately in 1985 and decreased again in 1997 (end of successive phase 3 of the KBP). The diversity of research topics was the highest in 1991 and 1992 and could be linked to KBP phase 2 for which the main objective was to conduct multidisciplinary research on plankton, reef ecology, fisheries, algae and dynamics of mangrove ecosystems.

### Taxonomic coverage

**Fig. 4:**
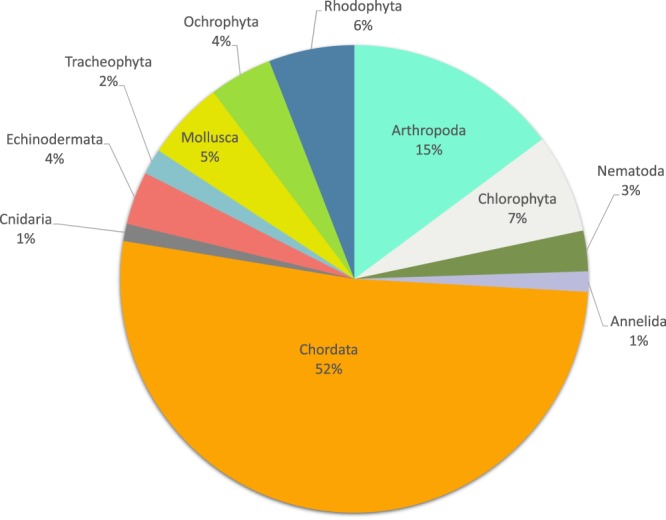
Taxonomic coverage of data collection: percentage of observations per phylum.

**Fig. 5:**
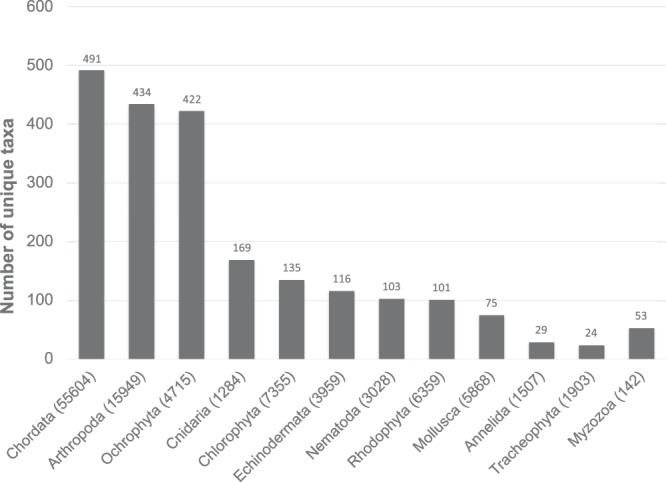
Taxonomic coverage of data collection: number of unique taxa per phylum.

99.8 % of original scientific names have been referenced through the WoRMS database representing in total 2,198 unique aphiaIDs. Within these unique aphiaIDs 78.8% of used names were accepted in literature, 18.2% was not accepted in literature, 1.5% were alternate representations (species that are represented twice: once with and once without subgenus) and 1.3% had an uncertain status (unassessed). 67.5% were identified to the species level (1490 unique aphiaID’s). 750 records were not suitable (group names e.g. meiobenthos, non-identified, eggs, zooplankton) for a taxonomic match in WoRMS. These records together with non-matches (0.2%) were not discarded. The unmatched names may be added to the register after approval of the dedicated editor. The total Kenya-Belgium data collection includes records for 29 different phyla (Fig. 4). Half of the observation records belonged to the Chordata (represented by birds and fish taxa) and although this frequency is almost 12 times higher than the Ochrophyta observations (4.3%), the number of unique taxa for both phyla were almost the same (Fig. 5). Remarkable are the 142 observations on Myzozoa listed in the database, as these represent 53 unique taxa. An important research topic within the Kenya-Belgium Project (KBP) was the description and inventory of benthic fauna in the mangroves of Gazi Bay. 15.8% of benthic invertebrate observations in Gazi Bay were identified at species level. The benthic invertebrate species community belonged to only 3 phyla and 6 classes, of which the Mollusca were most represented (57.7%). 30% of mollusc observations were on the mangrove oyster *Crassostrea cucullata*, and more specifically on its ecomorphology (physical properties of the shell and live biomass of observed species). Within the phylum of Arthropoda 2 classes were identified: Hexanauplia, represented by the benthic copepods (20 unique species), and Malacostraca of which all taxa belonged to the order of Decapoda, and more specifically to the Brachyuran mangrove crabs (56 unique species).

### Documentation and dataset dissemination

The Kenya-Belgium data collection^4^ is accessible through IMIS and can be downloaded from the Marine Data Archive (MDA).

## Technical Validation

The Kenya-Belgium database is a compilation of 68 biotic datasets which were digitized in the frame of a data rescue project. Focus was the digitization of historical biological data sampled in the Eastern African Region and/or in the frame of the Kenya-Belgium cooperation in marine sciences project (KBP) or collected by Belgian or Kenyan marine researchers. Many sources of historically collected data exist, but are scattered in different databases, hidden in papers or reports or even stored on old storage media such as cassettes, disks, tapes, cd’s, etc. There is an enormous amount of information already collected and stored in a standardized way about the world’s biodiversity. To date most of this old information has not been digitized. The digitization strategy used in this work was to stay as close to the original source data as possible. However, data from different sources always needs to be interpreted with caution. Hence, users of this dataset need to consider two caveats. First, not all data records hold data for all parameters/variables present in the final dataset, simply because they were not measured. Therefore, it might seem that there are quite some gaps. However, on the contrary, providing this information as well only increases the completeness of this dataset. Second, despite the effort to search the original publications for missing metadata, there are obviously still gaps (e.g. sampling date -and location) due to the fact that they were not properly described in the original publications. Notwithstanding the above caveats, the digitizing of historical data is crucial to fill in the spatial and temporal gaps in the data that is currently available to science. The current availability of biodiversity data from this region is still too low to give an adequate insight in the underlying processes that control the functioning of our ecosystems. This data rescue project certainly has improved and contributed to create a better accessibility (open access) and visibility of the data to the scientific community. The species list will be used to contribute to AfReMaS, the African Register of Marine Species - http://www.marinespecies.org/afremas), a taxonomic database of marine species found along the African coast. Next to that, these revitalized datasets can serve as a starting point for the biodiversity work in the Second International Indian Ocean Expedition (http://www.iioe-2.incois.gov.in) program. The sustainable use and management of biodiversity will require data about it to be available when and where decision-makers and scientists alike need that type of information.

## Usage Notes

All data are publicly available in Open Access and can be used under the license of CC BY with acknowledgement of the authors.

### ISA-Tab metadata file

Download metadata file

## Online-only Tables

**Online-only Table 1:** Overview of original sources digitized and integrated in the Kenya-Belgium data collection.

Author (publication date)	Source type	Data type
Aernaudts, H. (1990)	MSc Thesis	Biota
Anyango, C. (2000)	MSc Thesis	Biota/abiota
Bollen, A. (1993)	MSc Thesis	Biota/abiota
Boone, A. (1996)	MSc Thesis	Biota
Cattaert, S. (1997)	Thesis	Biota
De Bont, R. (1999)	Thesis	Biota
De Pauw, K. (1990)	MSc Thesis	Biota/abiota
De Schryver, T. (1990)	MSc Thesis	Biota/abiota
De Smet, I. (2000)	MSc Thesis	Biota
De Troch, M. (1995)	MSc Thesis	Biota
De Wit, M. (1988)	Ir Thesis	Biota/abiota
Demeulenaere, B. (1989)	BSc Thesis	Biota
Demeulenaere, B. (1991)	MSc Thesis	Biota
Dethier, K. (1997)	Thesis	Biota
Gallin, E. (1988)	Ir Thesis	Biota
Garcia de Camargo, M. (1995)	MSc Thesis	Biota/abiota
Geerinckx, T. (2002)	MSc Thesis	Biota
Gillikin, D.P. (2000)	MSc Thesis	Biota/abiota
Gurdebeke, S. (1998)	MSc Thesis	Biota/abiota
Humphreys, W.F. (1981)	Report	Biota
Joseck, D.M. (1991)	BSc Thesis	Biota
Juma, B. (1987)	Report	Abiota
Kairo, J.G. (1993)	MSc Thesis	Biota/abiota
Kazungu, J.M. (1986)	Report	Abiota
Leuci, D. (1995)	MSc Thesis	Biota
Luhunga, B.Y. (1997)	MSc Thesis	Biota
Melles, A. (1991)	MSc Thesis	Biota
Mutua, A.K. (2000)	MSc Thesis	Abiota
Muylle, J. (2000)	MSc Thesis	Biota
Mwamsojo, G.U.J. (1994)	MSc Thesis	Biota
Mwangi, S.N. (1994)	MSc Thesis	Biota/abiota
Nasirwa, O. *et al*. (1995)	Report	Biota
Ndede, R.O. (1998)	MSc Thesis	Biota
Ntiba, J.M. *et al*. (1991)	Report	Biota
Obade, P.T. (2000)	MSc Thesis	Biota/abiota
Okemwa, E. (1987)	Report	Biota
Okemwa, E. (1990)	PhD Thesis	Biota
Okemwa, E. *et al*. (1991)	Report	Biota
Okondo, J. (1995)	MSc Thesis	Biota/abiota
Osore, M.K.W. (1994)	MSc Thesis	Biota/abiota
Oteko, D. (1987)	Thesis	Abiota
Polk, P. (1986)	Report	Biota/abiota
Polk, P*. et al*. (1993)	Report	Biota/abiota
Poppe, B. (1997)	Thesis	Biota
Pratiwi, R. (1995)	MSc Thesis	Biota/abiota
Rao, R.G. (1991)	MSc Thesis	Biota
Reid, A. (2002)	MSc Thesis	Biota
Roels, T. (1998)	MSc Thesis	Biota
Ruwa, R.K. (1987)	Report	Biota
Samyn, Y. (1995)	MSc Thesis	Biota
Schallier, R. (1993)	MSc Thesis	Biota/abiota
Schrijvers, J. (1991)	Thesis summary	Biota/abiota
Schrijvers, J. (1996)	PhD Thesis	Biota/abiota
Silence, J. (1993)	BSc Thesis	Biota
Steyaert, M. (1993)	MSc Thesis	Biota/abiota
Vackier, I. (1993)	MSc Thesis	Biota
Van Avermaet, P. (1990)	BSc Thesis	Biota
Van den heede, C. (1994)	MSc Thesis	Biota
Van Hout, T. (1998)	MSc Thesis	Biota
Van Waeyenberge, J. (1994)	Thesis	Biota
Van Zele, M. (1992)	MSc Thesis	Biota
Vanhove, S. (1990)	MSc Thesis	Biota/abiota
Verellen, A. (1990)	MSc Thesis	Biota
Vermin, W. (1998)	BSc Thesis	Biota
Verneirt, M. (1994)	MSc Thesis	Biota/abiota
Verstraete, H. (1993)	MSc Thesis	Biota
Wakwabi, E.O. (1999)	PhD Thesis	Biota
Wakwabi, E.O. *et al*. (1991)	Report	Biota
Wamukoya, G. (1987)	Report	Biota
Wawiye, O. (1995)	MSc Thesis	Biota

**Online-only Table 2:** Temporal and geographical coverage of biota datasets integrated in the Kenya-Belgium data collection.

Dataset subject	Temporal coverage	Geographical coverage	No. records	Metadata link
Count of observed waterbirds	1995	Kenyan coast, Lake Victoria, Tana river	2235	https://doi.org/10.14284/175
List of microphytoplankton species	1992	Kenyan coast	228	https://doi.org/10.14284/74
List and counts of ichthyofauna of a seagrass bed	1993	Gazi Bay	31	https://doi.org/10.14284/113
List of phytal harpacticoid fauna	1986–1988	Gazi Creek, McKenzie Point, Fort Jesus	37	https://doi.org/10.14284/114
Counts and abundance of epiphytic meiofauna and nematodes from seagrasses	1989	Gazi, Nyali	1226	https://doi.org/10.14284/115
Counts of hyperbenthos	1994	Gazi Bay	610	https://doi.org/10.14284/79
Meiobenthos densities in *Avicennia marina* mangrove sediments	1992–1993	Gazi Bay	943	https://doi.org/10.14284/80
Abundance and habitat of echinoderm species	1969	Kenyan coast, Watamu, Mida Creek, Kilifi	1512	https://doi.org/10.14284/97
List of seagrasses and benthic macroalgae of the tidal channels	1989	Gazi Bay	9449	https://doi.org/10.14284/120
List of the genus Dictyopteris	1985–1999	Western Indian Ocean	70	https://doi.org/10.14284/96
List of Padina species	1985–1999	Western Indian Ocean	158	https://doi.org/10.14284/158
List of Cladophorales species	1985–1992	Kenyan coast	105	https://doi.org/10.14284/98
List of Codiales species	1985–1993	Kenya, Zanzibar, Seychelles	137	https://doi.org/10.14284/99
List of intertidal benthic diatoms	1991	Gazi Bay	273	https://doi.org/10.14284/81
List of Caulerpales species	1985–1989	Kenyan Coast	122	https://doi.org/10.14284/82
List of Siphonocladales species	1985–1992	Kenyan Coast	107	https://doi.org/10.14284/100
List of Ceramiaceae species	1985–1997	East-African coast	115	https://doi.org/10.14284/101
Qualitative abundance of zooplankton taxa	1985–1986	Port Reitz Creek	300	https://doi.org/10.14284/102
List of macrofauna species	1986	Gazi, Kanamai, Malindi Bay	119	https://doi.org/10.14284/103
List of Dictyotales species	1985–1994	Kenya, Tanzania	107	https://doi.org/10.14284/107
List of seagrasses and associated macroalgae	1987	West Gazi Bay	380	https://doi.org/10.14284/117
Counts of epibenthic fish species	1996	Gazi Bay	410	https://doi.org/10.14284/94
Composition and densities of the meiobenthic community	1996	Gazi Bay	6057	https://doi.org/10.14284/85
Counts of Mysidacea sp.	1994	Gazi Bay	53	https://doi.org/10.14284/87
Counts of Isopoda	1994	Gazi Bay	97	https://doi.org/10.14284/83
Counts of Mysidacea sp.	1994–1996	Gazi, Uroa, Ambon	20	https://doi.org/10.14284/84
List of sampled reef corals	1990	Kenyan Coast	82	https://doi.org/10.14284/109
Shell measurements and biomass of the mangrove oyster *Crassostrea cucullata*	1991	Gazi	133	https://doi.org/10.14284/86
List of Typhloplanoida	1985–1997	Zanzibar, Kenya, Kerguelen, Australia, Weddell Sea	42	https://doi.org/10.14284/93
Counts of *Echinothrix diadema* and its substrate coverage	1997	Mombasa Marine National Park and Reserve	66	https://doi.org/10.14284/89
List of harpacticoid copepods in tropical seagrass beds	1996	Gazi Bay, Mbweni	164	https://doi.org/10.14284/108
Length, weight measurements of *Sphaeramia orbicularis*, counts of potential prey items and other observed fish	1993	Gazi Bay	330	https://doi.org/10.14284/75
List, counts and length of observed fish	1990	Gazi Creek	31	https://doi.org/10.14284/111
Zooplankton taxon abundances	1990–1991	Gazi Creek	870	https://doi.org/10.14284/110
Epibenthos and fish species counts	1994	Gazi Bay	7868	https://doi.org/10.14284/95
Length, width and weight of *Echinotrix diadema* and *E. Calamaris* species	1994–1995	Mombasa Marine National Park	424	https://doi.org/10.14284/88
Overview and counts of phytal Harpacticoida collected on seagrasses	1991	Gazi Bay, Tudor Creek	338	https://doi.org/10.14284/105
List of zooplankton taxa and copepod species	1984–1987	Tudor Creek	1003	https://doi.org/10.14284/118
List of epiphytic macroalgae species and coverage percentages	1989	Gazi Bay	3839	https://doi.org/10.14284/119
Cell measurements and densities on phytoplankton species	1989	Gazi Creek	54	https://doi.org/10.14284/142
Zonation and list of mangrove vegetation and associated fauna	1993	Gazi Bay, Mida Creek	365	https://doi.org/10.14284/150
Abundance of *Asterropteryx semipunctatus* and *Gnatholepis sp.1*	1995	Gazi Bay	222	https://doi.org/10.14284/77
Density of mangrove meiobenthos and nematode biomass measurements	1989	Gazi Bay	2453	https://doi.org/10.14284/144
Zooplankton densities	1998	Shirazi bay	810	https://doi.org/10.14284/91
Qualitative abundance data on marine flora	1986–1987	Kenyan Coast	1980	https://doi.org/10.14284/106
Counts and size ranges of fish species	1990–1991	Gazi Creek	71	https://doi.org/10.14284/112
Counts of observed fish species	1993	Gazi Bay	750	https://doi.org/10.14284/146
Benthos densities	?	Gazi Bay	10	https://doi.org/10.14284/145
Catch data on fish and invertebrates	1994–1996	Gazi Bay	55151	https://doi.org/10.14284/152
Zooplankton and phytoplankton densities in oyster farms	1992	Gazi Creek	1770	https://doi.org/10.14284/149
Copepoda species list and zooplankton densities	1990–1992	Gazi, Lamu, Malindi	2857	https://doi.org/10.14284/153
Macrobenthos densities and nematode biomass sampled in a *Ceriops tagal* mangrove	1992–1993	Gazi Bay	1326	https://doi.org/10.14284/155
List of found macroalgal and seagrass species and study on algal community on pneumatophores of mangrove trees	1987	Gazi Bay	601	https://doi.org/10.14284/164
Counts of mangrove crabs in mangrove forests	1998–1999	Dabaso, Gazi	46	https://doi.org/10.14284/157
Mangrove species list	?	Gazi Bay	8	https://doi.org/10.14284/179
Species composition and biomass of seagrass and macroalgae communities	1991	Gazi Bay	119	https://doi.org/10.14284/163
List of epiphytic macroalgae and relative abundance of mangrove microphytobenthos	1987–1991	Gazi Bay	65	https://doi.org/10.14284/159
Zooplankton qualitative abundances and species composition	1990–1991	Gazi Creek	111	https://doi.org/10.14284/161
Density of major macrobenthos taxa in mangroves	1990	Gazi Bay	480	https://doi.org/10.14284/162
Abundance and length measurements of identified fish species	1991–1992	Gazi Creek	399	https://doi.org/10.14284/165
Checklist of hermatypic coral species and distribution data of coral genera	?	Kenyan Coast	364	https://doi.org/10.14284/166
Relative abundances, counts and length measurements of penaeid shrimps	1986	Tudor Creek	91	https://doi.org/10.14284/167
List of estuarine fish species	1985	Port Reitz, Tudor Creek	33	https://doi.org/10.14284/176
Presence/absence data on brackish water crabs	1985–1986	Gazi, Kanamai, Bamburi, Mkomani	112	https://doi.org/10.14284/168
Presence/absence data on planktonic copepods	1984–1985	Tudor Creek	159	https://doi.org/10.14284/171
Zooplankton abundance and dry weight and length measurements of copepods	1985–1986	Fort Jesus, Tudor Creek	37	https://doi.org/10.14284/172
Identification and habitat description of *Gracilaria salicornia* and other algae	1986	MacKenzie Point	67	https://doi.org/10.14284/177
Presence/absence of macroalgae and their zonation patterns on rocky cliffs	1985	Mombasa	1182	https://doi.org/10.14284/178
